# “Good Wine Makes Good Blood”: An Integrated Approach to Characterize Autochthonous Apulian Grapevines as Promising Candidates for Healthy Wines

**DOI:** 10.7150/ijbs.70287

**Published:** 2022-04-11

**Authors:** Wilma Sabetta, Mariangela Centrone, Mariagrazia D'Agostino, Graziana Difonzo, Luigi Mansi, Giovanni Tricarico, Pasquale Venerito, Ernesto Picardi, Luigi Ruggiero Ceci, Grazia Tamma, Francesco Caponio, Cinzia Montemurro, Mariateresa Volpicella

**Affiliations:** 1 Institute of Biosciences and BioResources (IBBR), National Research Council (CNR), Via Amendola 165/A, 70126 Bari, Italy.; 2 Spin off Sinagri s.r.l., University of Bari Aldo Moro, Via Amendola 165/A, 70126 Bari, Italy.; 3 Department of Biosciences, Biotechnologies and Biopharmaceutics, University of Bari Aldo Moro, Via Amendola 165/A, 70126 Bari, Italy.; 4 Department of Soil, Plant and Food Sciences, University of Bari Aldo Moro, Via Amendola 165/A, 70126 Bari, Italy.; 5 Confcooperative Puglia, Viale Einaudi 15, 70125 Bari, Italy.; 6 CRSFA-Centro Ricerca, Sperimentazione e Formazione in Agricoltura “Basile Caramia”, Via Cisternino, 281, 70010 Locorotondo (BA), Italy.; 7 Institute of Biomembranes, Bioenergetics and Molecular Biotechnologies (IBIOM), National Research Council (CNR), Via Amendola 165/A, 70126 Bari, Italy.; 8 Institute for Sustainable Plant Protection-Support Unit Bari (IPSP), National Research Council (CNR), Via Amendola 165/A, 70126 Bari, Italy.

**Keywords:** Apulian grapevines, Negramaro, Nero di Troia, Susumaniello, transcriptomics, anthocyanin pathway, wine composition, antioxidant effects

## Abstract

Wine production represents an ancient human activity and one of the most economically important markets in Europe. Moreover, the health effects of grapes and related products have been largely demonstrated, and mostly depend on their richness in bioactive molecules such as flavonoid and non-flavonoid phenolic compounds. Italy has the highest global wine production and provides one of the richest grapevine germplasm in the Mediterranean area. In this paper, our attention was focused on the evaluation of the total phenol and anthocyanin content in five autochthonous Apulian grapevine cultivars, in both wines and their non-alcoholic extracts. Moreover, the potential antioxidant effects of the non-alcoholic wine extracts on the cell viability of Caco-2 and HeLa carcinoma cell lines were tested. Finally, for the most promising autochthonous selected cultivars (Negramaro, Nero di Troia and Susumaniello), comparative transcriptomic analysis in berries was performed using high-throughput sequencing technology.

## Introduction

Grapevine (*Vitis spp*) is one of the most economically important and widely cultivated fruit crops all over the world, with a well-established wine market in Europe. Currently, the global annual production of grapes is over 75 million tones, almost the 50% is used for the wine industry, and the other part is used for fresh consumption as table grapes or for making jam, grape juice, jelly, grape seed extract, raisins, vinegar and grape seed oil (http://www.wineinstitute.org). In this context, Italy has the highest global wine production (19% of the total) with over 47 million hL, with 671 thousand hectares dedicated to viticulture, 46 thousand winemaking companies, and 13 billion € of total turnover (OIV - Organisation Internationale de la Vigne et du Vin, 2020, Wine Production - OIV First Estimates). Moreover, due to their typicality brands, about 50% of Italian wines are certified products with PDO (Protected Designation of Origin), PGI (Protected Geographical Indication), and DOCG (Designation of Controlled and Guaranteed Origin) marks (EEC Regulation No. 2081/92).

The health effects of grapes and related products have been largely assessed and demonstrated 1-3. They are mostly related to the grape richness in bioactive molecules, including flavonoid compounds (flavonol, monomeric catechins, pro-anthocyanins, anthocyanins, anthocyanidins) and non-flavonoid phenolic compounds (resveratrol) together with other nutrients, metabolites and vitamins 4. Notably, flavonoid compounds have a critical role both on the wine quality, by contributing to its bitterness and astringency (pro-anthocyanidins), and on the color (anthocyanins). Moreover the beneficial effects on human health are related to the prevention of oxidative reactions, inflammatory processes, degenerative pathophysiological state in adults, and cardiovascular diseases 2,5-8. Recent studies also demonstrated the fundamental role of red wine polyphenols in reinforcing and protecting the intestinal barrier against inflammatory stimulus by affecting the tight junction protein expression 9. However, the large part of scientific publications commonly reports about international and well known grape cultivars 10-12, while little information is currently available about the content of these compounds in minor cultivars.

In the last few years, Next Generation Sequencing (NGS) technologies provided several high-throughput transcriptomic profiles in the most common *V. vinifera* cultivars and contributed to creating a comprehensive picture of gene regulation in this species, showing the complex and distinct biochemical pathways active in seeds, skin, and pulp during berry development 13-19. To date, little is known about gene expression in other *Vitis* cultivars and particularly in the minor autochthonous ones.

**Figure 1:**
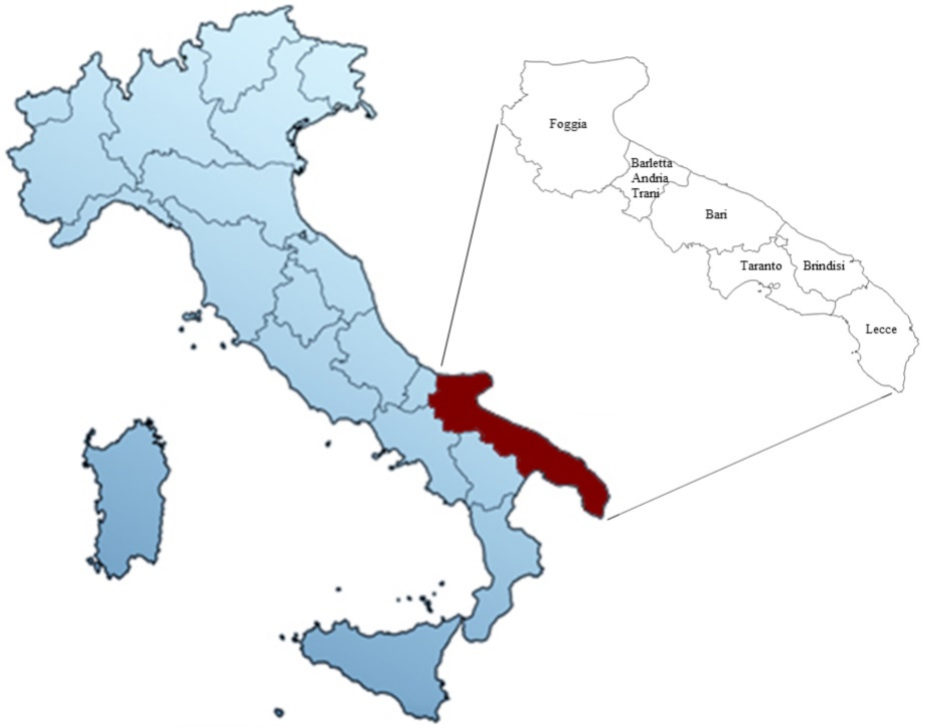
Map of Italy with particular focus on the Apulia region and its provinces.

Here for the first time, three major and well known Italian cultivars (Negramaro, Nero di Troia and Primitivo) and two minor autochthonous red cultivars (Notardomenico and Susumaniello) of the Apulia region (Southern Italy, Fig. 1) were selected to study possible anti-oxidative effects of their wine extracts and the correlated association with distinct transcriptomic profiles. Apulia is the second Italian region, after Veneto, with a rich heritage of grape genetic resources 20,21, deriving from about 86 thousand of wine-growing acreage, with over 9 million hL production, 2% PDO, and 19% PGI wines (versus 38% and 13% of Veneto, respectively) (ISMEA - Istituto di servizi per il mercato agricolo, 2021). In recent years, Apulia has undergone an important process of valorization and qualification of its wines, leading to a huge diffusion and appreciation on the national and international markets too 22. Indeed, the export value of Apulian wines in 2020 increased to 164 million euros, especially thanks to the markets of Germany, UK, and USA.

Negramaro, a non-aromatic red wine grape cultivar, is originally from the Southern part of Apulia, but it is grown all over the region. The Negramaro wine presents a dark red color, with a balanced organoleptic bouquet aroma. It is traditionally used in combination with other grape varieties to produce 14 Apulian PDO wines. Nero di Troia wine is made from the autochthonous regional grape variety Uva di Troia, a common denominator of several wines produced in North-Apulia. It has a ruby red color with orange reflections, neutral flavor, high alcohol and tannin contents, but a very low acidity level. For this reason, it is preferably vinified in combination with other varieties thus obtaining several PDO, DOCG, and PGI Apulian wines, even if recent oenological advances are making possible to produce prestigious Uva di Troia mono-varietal wines. It is mostly used for red vinification and only recently, to produce rosé wines. Primitivo is the most diffused and cultivated grape variety in the Apulia region as well as in Southern Italy, especially in areas with less favorable climate; it is an early, strong wine cultivar able to produce bunches on secondary shoots too, 15-20 days later the first ripening. Primitivo wine is characterized by high alcoholic and tannic notes, with a ruby-purple color and a spicy, red-fruit aroma, that makes it suitable for blending with weaker wines and for the production of all Apulian PGI and several PDO and DOCG wines. Notardomenico and Susumaniello are part of the minor Apulian autochthonous cultivars. The first is mostly diffuse in the province of Brindisi and more recently in other areas of the region, although the cultivation surfaces are rather modest. Notardomenico wine is ruby red color with violet reflections, aromatic and spicy notes, and hints of ripe fruits. Its alcoholic and anthocyanin contents make it suitable for producing pure rosé wines or, more commonly, blended with other indigenous varieties. Originating from the province of Brindisi, Susumaniello is currently widespread in other areas of Apulia and Southern Italy too, including the Calabria region, where it is known with different names 23,24. It was initially famous for its strong coloring power, but very recently its aptitude for red and rosè vinification has been rediscovered and utilized for the production of 2 PDO and 5 PGI wines.

In this paper, considering the five mentioned Apulian grape cultivars, we first describe the total phenol and anthocyanin contents in wines and their non-alcoholic extracts. Subsequently, the potential antioxidant effects of the non-alcoholic wine extracts on the cell viability of Caco-2 and HeLa carcinoma cell lines were tested. Moreover, to gain more insight into the molecular signals activated by the extracts, the phosphorylation levels of p38-MAPK and NFkB, a crucial mediator of the inflammatory response, were evaluated as well. Following, to gain information on genes controlling anthocyanin accumulation, comparative transcriptomic analysis in the berries of the most promising cultivars Negramaro, Nero di Troia and Susumaniello was performed using a high-throughput sequencing technology.

## Material and methods

### Plant material and berries sampling

Five black-skinned autochthonous Apulian grape cultivars, such as Negramaro (NA), Nero di Troia (NT), Notardomenico (N), Primitivo (P), Susumaniello (SM), were grown in the experimental field of CRSFA Institute (Centro di Ricerca, Sperimentazione e Formazione in Agricoltura “Basile Caramia”) located in Locorotondo (Bari, Southern Italy). Berry samples from each cultivar were manually harvested when fully ripe, according to the optimal sugar content (22-25 °Brix), total acidity (0.6-0.8%) and pH values (3.2-3.4) for vinification and commercial use. For transcriptomic analysis, 6 different berries were randomly collected from the same plant and immediately frozen in liquid nitrogen. For each sample, three biological replicates were taken, and stored at -80 °C until use.

### Vinification and non-alcoholic extracts

Monovarietal wines from the autochthonous selected grape cultivars were produced in the laboratories of the experimental wine cellar of CRSFA Institute by using a standard micro-vinification protocol with some modifications, in order to obtain wines with high antioxidant content, according to the Method OIV-VITI 609-2019 reported into the Compendium of International Methods of Analysis (https://www.oiv.int/public/medias/6944/oiv-viti-609-2019-it.pdf). In particular, about 80-100 kg of grapes from three biological replicates of each cultivars were refrigerated at 4-6 °C for 24 h, destemmed with the addition of 8 g/hL of potassium metabisulphite, cryo-macerated for 24 h at 6 °C and let warm up to 20 °C. Ammonia salts with 10 g/hL thiamine and selected yeasts (*Saccharomyces cerevisiae*) were added, and the whole mass was punched down 2 times per day during fermentation. At the stage of 4-5 alcohol degrees, the whole mass was oxygenated and treated with ammonia salts with 15 g/hL thiamine. When 9 alcohol degrees were reached, ammonia salts with 5 g/hL thiamine were added. The mass was pressed at the complete exhaustion of sugars, and wines were statically clarified at 0 °C for one week, then transferred in steel tanks and let to naturally warm up to 20 °C. Malolactic fermentation was then favored by using lactic acid bacteria (*Oenococcusoeni*). After racking, wines were poured and SO_2_ was added to a final content of 25 mg/L free SO_2_. Wine refinement and stabilization with controlled oxygenation were monitored every 15 days. Finally, the obtained wines were stored at 16-18 °C, bottled after 3 months, and then analyzed.

Specifically, the non-alcoholic extracts were obtained from wines through evaporation (Rotavapor, Buchi) setting the temperature at 40 °C and reaching the final pressure of 2 mbar. The evaporation was carried on until a reduction in the volume of 35% was achieved.

### Chemical characterization of wines and non-alcoholic extracts

Wines and non-alcoholic extracts obtained from each selected cultivars were characterized for total phenol content and anthocyanin profile. Three biological replicates were considered for this analysis. The determination of total phenol content (TPC) was performed by the Folin-Ciocalteu method according to Difonzo et al. 25 with some modifications. In particular, 20 µL of wine or extract were added to 980 µL of ddH_2_O and 100 µL of Folin-Ciocalteu reagent. After 3 min, a 5% Na_2_CO_3_ solution was added and incubated at room temperature for 60 min. The absorbance was read at 750 nm using a Cary 60 spectrophotometer (Agilent, Cernusco, Milan). The TPC was expressed as gallic acid equivalents (GAE) in mg/L of extract.

The anthocyanin profile was determined by HPLC-DAD according to Tarantino et al. 26 and Gambacorta et al. 27 with some modifications. The extracted samples were filtered through a 0.45 µm size nylon membrane filter and placed in a 1.5 mL vial in an automatic sampler for injection. The HPLC analysis of the phenolic extracts was performed using a Thermo Scientific HPLC system (Dionex, Germering, Germany) equipped with a WPS-3000 RS autosampler, an HPG-3200 RS pump, TCC-3000 column compartment, and an L-2450 diode array detector. The separation was carried out with an analytical column RP-C18 column Acclaim^TM^ 120 (Thermo Scientific, 3 µm particle size, 120 Å pore size, 150 × 3.0 mm) at 30 °C. The diode array detector was set at an acquisition range of 200-600 nm. The identification of phenolic compounds was performed by comparing the peak retention times with those obtained by the injection of pure standards and, in absence of these, with data in the literature 27. The quantification was performed by using an external calibration curve made with malvidin-3-glucoside.

### Cell assays

#### Cell culture and treatment

The human colon cancer cells (Caco-2) were grown in Minimum Essential Medium (MEM), obtained from BIOWEST (BIOWEST, Riverside, CA, USA), supplemented with 20% fetal bovine serum (FBS), 100 i.u./mL penicillin, 100 µg/mL streptomycin at 37 °C in 5% CO_2_. Alternatively, human cervical cancer cells (HeLa) were grown in MEM supplemented with 10% fetal bovine serum (FBS), 100 i.u./mL penicillin, 100 µg/mL streptomycin at 37 °C in 5% CO_2_. Cells were left under basal condition or treated for 2 h with 300 mg/L GAE of non-alcoholic extracts of NA, NT, N, P, and SM cultivars. Three biological replicates were considered in this analysis too.

#### Crystal violet assay

Crystal violet assay was performed as previously described 28. Cells were grown in a 96-well plate and left under basal condition or stimulated as mentioned before, and then fixed with 4% paraformaldehyde in phosphate-buffered saline (1 X PBS) for 20 minutes, washed in PBS, and stained with a solution containing 0.1% crystal violet in 20% methanol for 20 min. After washing, cells were lysed with 10% acetic acid. As a measurement of cell viability, the optical density at 595 nm (OD_595_) of each well was measured with a Microplate Reader (Bio-Rad Laboratories, Inc., Hercules, CA, USA).

#### ROS detection

Reactive Oxygen Species (ROS) were detected as already reported 29,30. After treatments, cells were incubated with dihydrorhodamine-123 (10 μM) for 30 min at 37 °C, with 5% CO_2,_ and recovered in complete medium for 30 min. As a positive control, cells were treated with *tert*-Butyl hydroperoxide (tBHP, 2 mM for 30 min). Cells were lysed in RIPA buffer pH 7.4 containing 150 mM NaCl, 10 mM Tris-HCl pH 7.2, 0.1% SDS, 1% Triton X-100, 1% sodium deoxycholate and 5 mM EDTA pH 8. Samples were then collected and centrifuged at 12,000 × *g* for 10 min at 4 °C and the supernatants were used for ROS detection. The fluorescence emission signal was recorded using a fluorimeter (RF-5301PC, Shimadzu Corporation, Kyoto Japan) at excitation and emission wavelengths of 508 and 529 nm, respectively.

#### Immunoblotting

Cells were seeded onto 60-mm dishes and were left under basal or treated conditions as described in “*Cell culture and treatment”* section*.* After their lysis with ice-cold RIPA buffer in the presence of proteases (1 mM PMSF, 2 mg/mL leupeptin, and 2 mg/mL pepstatin A) and phosphatases (10 mM NaF and 1 mM sodium orthovanadate) inhibitors, suspensions were centrifuged at 12,000 × *g* for 10 min at 4 °C. Obtained supernatants were collected and 60 μg of proteins were separated on 10% or 12% stain-free polyacrylamide gels (Bio-Rad Laboratories, Inc., Hercules, CA, USA) under reducing conditions. Protein bands were electrophoretically transferred onto Immobilon-P membranes (Merck KGaA, Darmstadt, Germany) for Western blot analysis. The membrane was blocked in TBS-Tween-20 containing 3% bovine serum albumin (BSA) and incubated overnight at 4 °C with primary antibodies, anti-phospho-NF-kB p65 and anti-NF-kB p65 purchased from Santa Cruz Biotechnology (Santa Cruz Biotechnologies, Dallas, TX, U.S.A.), and anti-p38-MAPK and anti-phospho-p38-MAPK (Thr180/Tyr182) purchased from Cell Signaling Technology (Cell Signaling Technology, Leiden, The Netherlands). Immunoreactive bands were detected with horseradish peroxidase-coupled secondary antibodies, goat anti-rabbit antibody purchased from Merck (Merck KGaA, Darmstadt, Germany), and secondary goat anti-mouse antibodies obtained from Bio-Rad (Bio-Rad Laboratories, Inc., Hercules, CA, USA). Membranes were incubated with Super Signal^®^ West Pico Chemiluminescent Substrates (Thermo Fisher Scientific, Waltham, MA, USA) and chemiluminescence was detected with the ChemiDoc System (Bio-Rad Laboratories, Inc., Hercules, CA, USA). Obtained bands were normalized to total protein using the stain-free technology gels. Densitometry analysis was performed using Image Lab (Bio-Rad Laboratories, Inc., Hercules, CA, USA) and data were analyzed and summarized in histograms with GraphPad Prism (GraphPad Software, San Diego, CA, USA).

### Transcriptome and gene expression analysis

#### Purification of total RNA for high throughput sequencing

For each selected cultivar three biological replicates were used. Six frozen berries randomly collected from each plant were mechanically crushed by using a tissue lyser and total RNA was extracted from 400 mg of the obtained powder using the Spectrum Plant Total RNA Kit (Sigma-Aldrich, St. Louis, MO, USA), as already reported 31. An on column DNAse I (Sigma-Aldrich, St. Louis, MO, USA) treatment was included according to manufacturer's instructions. A further purification step was then performed by adding an appropriate volume of 8 M lithium chloride solution (Sigma-Aldrich, St. Louis, MO, USA) to obtain a final concentration of 3 M. After overnight incubation at 4 °C, the RNA samples were harvested by centrifugation at 13,000 × *g* for 20 min at 4 °C. Pellets were washed with 70% cold ethanol, centrifuged for 5 min at 13,000 × *g* at 4 °C, and completely dried. Purified pellets were finally diluted in 40 µL of elution buffer (Sigma-Aldrich, St. Louis, MO, USA). RNA quality and quantity were checked by spectrophotometric measurement using a Nanodrop 2000 spectrophotometer (Thermo Scientific, Waltham, MA, USA) and by electrophoresis on 1.2% Certified Molecular Biology Agarose gel according to consolidated procedures 32.

#### cDNA library construction and transcriptome sequencing

On the basis of cell assay results, cDNA libraries were synthesized from three biological replicates of the selected NA, NT and SM grape cultivars. A total amount of 2.5 μg RNA per sample was used for library preparation, using Illumina's TruSeq Stranded Total RNA Sample Preparation Kit (Illumina, San Diego, CA, USA), according to the manufacturer's protocol. Libraries were then checked on the Bioanalyzer 2100 and quantified by fluorimetry using the Quant-iTTMPicoGreen^®^ dsDNA Assay Kit (Thermo Fisher Scientific, Waltham, MA, USA) on NanoDrop™ 3300 Fluorospectrometer (Thermo Fisher Scientific, Waltham, MA, USA). Sequencing was performed on NextSeq 500 platform, generating on average 38 million of 100 bp paired-end reads per sample.

#### RNAseq data analysis

RNAseq reads in FASTQ format were initially inspected using the FastQC program (http://www.bioinformatics.babraham.ac.uk/projects/fastqc). Adaptor sequences and low quality regions (phred cutoff of 25) were trimmed using fastp (version 0.20.0) (with parameters: --detect_adapter_for_pe -x -q 25 -n 1 -l 50 -y -w 8), retaining only reads with a minimal length of 50 bases (Tab. S1) 33. Next, cleaned reads were aligned onto the complete *V. vinifera* genome (assembly version GCA_000003745.2, downloaded from ftp://ftp.ensemblgenomes.org/pub/plants/release-47/fasta/vitis_vinifera/dna/) using STAR (version 2.5) with default parameters and providing a gtf file with known annotations (downloaded from ftp://ftp.ensemblgenomes.org/pub/plants/release-47/gff3/vitis_vinifera) 34. Read counts per gene were performed by Feature Counts (version1.6.0) with default parameters for paired-end reads and considering the stranded nature of sequences (Tab. S2) 35. Differential gene expression was carried out using DESeq2 (version1.28.1) 36. Only genes with an adjusted *p* value <0.05 and |log_2_(FC)|>1 were taken into account for downstream analyses. Sequencing raw data are available in the SRA (Short Read Archive) database under the BioProject PRJNA799026.

Gene ontology on unique genes and differentially expressed genes (DEGs) for each cultivar was performed using agriGO (http://bioinfo.cau.edu.cn/agriGO/) 37 and results were analyzed in rStudio and displayed by dot-plots. DEGs linked to the grapevine anthocyanidin metabolism were investigated at pathway level using KEGG pathway (http://www.genome.jp/kegg/pathway.html) 38, UNIPROT (https://www.uniprot.org/) and ENSEMBLPlants (http://plants.ensembl.org/index.html) databases.

#### Quantitative real-time PCR (qRT-PCR)

To validate transcriptomic data, the expression level of selected genes was evaluated by qRT-PCR analysis. One μg RNA aliquots of the selected NA, NT, and SM cultivars were reverse transcribed using the SuperScript™ VILO™ cDNA Synthesis Kit (ThermoFisher, Waltham, MA, USA) according to the manufacturer's instructions. qRT-PCR reactions were performed using the Sso Advanced Universal SYBER^®^ Green Supermix (Bio-Rad Laboratories, Hercules, California, USA) and the CFX96 Touch Real-Time PCR Detection System (Bio-Rad Laboratories, Hercules, California, USA). Thermal cycling parameters were: initial denaturation at 95 °C for 3 min, followed by 40 cycles of 95 °C for 10 s and 60 °C for 30 s. The existence of a unique PCR product was confirmed by evaluating the “melting curve” through an increase of 0.2 °C every 5 s from 65 to 95 °C. All analyses were conducted on the same RNA aliquots already used for NGS sequencing and samples from the same cDNA were run in triplicate, setting the intra-assay repeatability between technical replicates below 0.5 Ct. The elongation factor 1γ (EF1γ, AF176496.1) was used as a reference gene for normalization and the comparative Ct method (2^-ΔΔCt^ method) was used to analyze the expression levels of the selected genes 39. All the primer sequences regarding reference and target genes are reported in the Supplementary Material (Tab. S3).

Differences between cultivars in qRT-PCR experiments were assessed using unpaired Student's t-test using SigmaPlot software v. 12.2 (Systat Software Inc., Canada, USA).

#### Statistical analysis

Experimental analyses data were compared reporting the mean values (n=2 or n=3) ± standard deviation (sd). Data obtained for the chemical characterization of wines and non-alcholic extracts were analysed using the ANOVA and Tukey's test by MATLAB^®^ statistics toolbox (The Mathworks Inc., Natick, MA, USA). *In vitro* experiments were shown as mean ± standard error of the mean (SEM) and the statistical analysis was performed using One-way ANOVA followed by Dunnett's multiple comparisons test using GraphPad Software (San Diego, USA). Differences with *p* < 0.05 were considered statistically significant.

## Results and Discussion

### Wine and non-alcoholic extract composition and characterization

The values of the main chemical parameters obtained from wine characterization are shown in Table 1, and resulted in agreement with those reported in the literature for different Apulian red wines 27,40. The highest values of pH, alcohols, sugars, ashes, K^+^, and dry residue extract were found in P cultivar. No relevant differences among cultivars were detected for the other parameters, despite N cultivar showed the highest value of titratable acidity.

The mono-varietal non-alcoholic extracts obtained from wines were characterized for the total phenol content (TPC) and the anthocyanin composition (Table 2). Concerning TPC, the differences found among the extracts were in line with the literature, since the phenol extraction from grapes seems to be mostly variety-dependent 41,42. The highest content of phenolic compounds was observed in P and NT cultivars. Anthocyanins are the most abundant phenols in red grape skins and they are directly responsible for wine color, exerting at the same time bioactive properties 8. As expected, their concentration showed differences among the non-alcoholic extracts, confirming that the anthocyanin profile in grapes is strictly related to the cultivar 43. In particular, SM was found as the richest cultivar in anthocyanins, followed by NA, whereas the lowest concentrations of the identified compounds were found in P and NA cultivars (Table 2). In all samples, malvidin-3-glucoside was the anthocyanin with the highest level followed by petunidin-3-glucoside. In fact, the most abundant compounds found in the grape red wines are malvidin derivatives and the main monomeric anthocyanins are the 3-*O*-monoglucosides and malvidin 3-*O*-glucoside. Derivatives of malvidin 3-*O*-glucoside are usually the most abundant component and represent the main source of the red color in very young red wines 44, although recent studies highlight the importance of different phenomena on color and stabilization of anthocyanins, such as the co-pigmentation with phenolic compounds in wine 45.

### *In vitro* effects of non-alcoholic wine extracts

**Figure 2:**
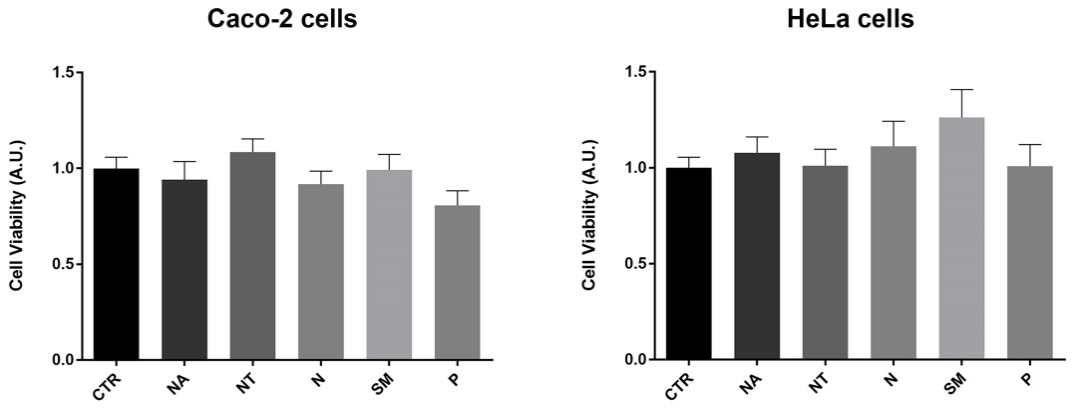
** Cell viability in Caco-2 and HeLa cells.** Data are shown as means±SEM of 12 independent experiments and analyzed by One-way ANOVA followed by Dunnett's multiple comparisons test. Abbreviations: CTR, untreated conditions; NA, Negramaro; N, Notardomenico; P, Primitivo; SM, Susumaniello; NT, Nero di Troia.

Several studies provide evidence that a moderate red wine consumption has beneficial properties for human health. In particular, polyphenols contribute to decrease the risk of endothelial and cardiovascular dysfunctions, hypertension, dyslipidemia, and metabolic disorders 46. However, several phytocompounds known for their antioxidant properties may instead have a cytotoxic and pro-oxidant effect 47. In human erythrocytes, red wine polyphenols displayed a pro-oxidant effect that stimulates a compensative antioxidant defense response 48. Here, the potential effects of the non-alcoholic wine extracts on cell viability were investigated using the crystal violet assay. Caco-2 and HeLa cells were exposed for 2 hours to 300 mg/L GAE of non-alcoholic extracts of all the selected Apulian grapevine cultivars. Compared to untreated conditions (CTR), treatments with all the non-alcoholic extracts did not alter cell viability at the concentration used here (Fig. 2).

**Figure 3:**
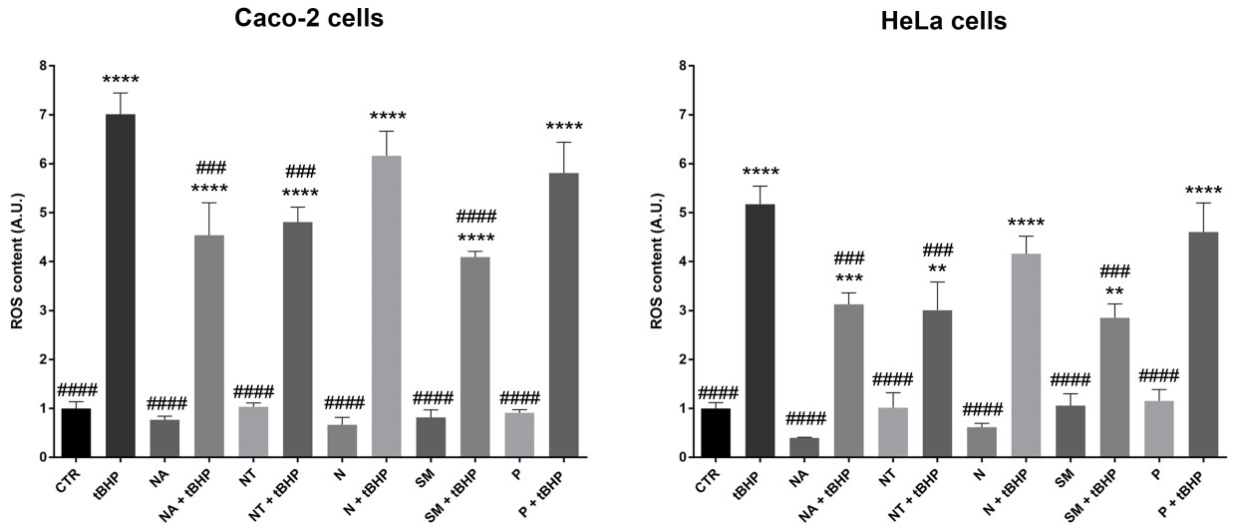
** ROS content in Caco-2 and HeLa cells.** Cells were left under basal conditions or exposed for 2 hours to 300 mg/L GAE of non-alcoholic extracts; ROS content was measured using dihydrorhodamine-123 fluorescence. Data are shown as means±SEM of 4 independent experiments and analysed by One-way ANOVA followed by Dunnett's multiple comparisons test (****p<0.0001,***p<0.001 and **p<0.01 *vs* CTR; ####*p*<0.0001, ###*p*<0.001, ##*p*<0.01 and #*p*<0.05 *vs* tBHP). Abbreviations: CTR, untreated conditions; tBHP, *tert*-Butylhydroperoxide; NA, Negramaro; N, Notardomenico; P, Primitivo; SM, Susumaniello; NT, Nero di Troia.

The total antioxidant ability of red wine is mainly due to the free anthocyanins fraction that is involved in the electron transfer mechanism. Indeed, free anthocyanins display a hydroxyl radical scavenger activity 48. Here, the antioxidant ability of selected wine extracts on the carcinoma cell lines was tested. Interestingly, compared to the cells treated with the oxidant *tert*-Butyl hydroperoxide (positive control), the treatment with the non-alcoholic extracts from SM, NT, and NA wines significantly reduced ROS levels induced by tBHP (Fig. 3). Incubation with the non-alcoholic extracts alone does not alter ROS content. These findings, indeed, revealed that the quantitative and the qualitative composition of the extracts, in terms of polyphenols, plays a key role in the oxidative response. In an endothelial model of insulin resistance and hyperglycemia, Magliocco wine, which contains a higher level of malvidin compared with Gaglioppo and Nerello wines, exerted the highest antioxidant ability 49. Interestingly, N and P that have a slightly lower content of malvidin 3-glu, did not reduce the tBHP-induced ROS generation. By contrast, SM that has the highest content of malvidin, significantly reduced the intracellular ROS content in both carcinoma cell lines.

### Effects of non-alcoholic extracts on NF-kB and p38-MAPK phosphorylation

**Figure 4:**
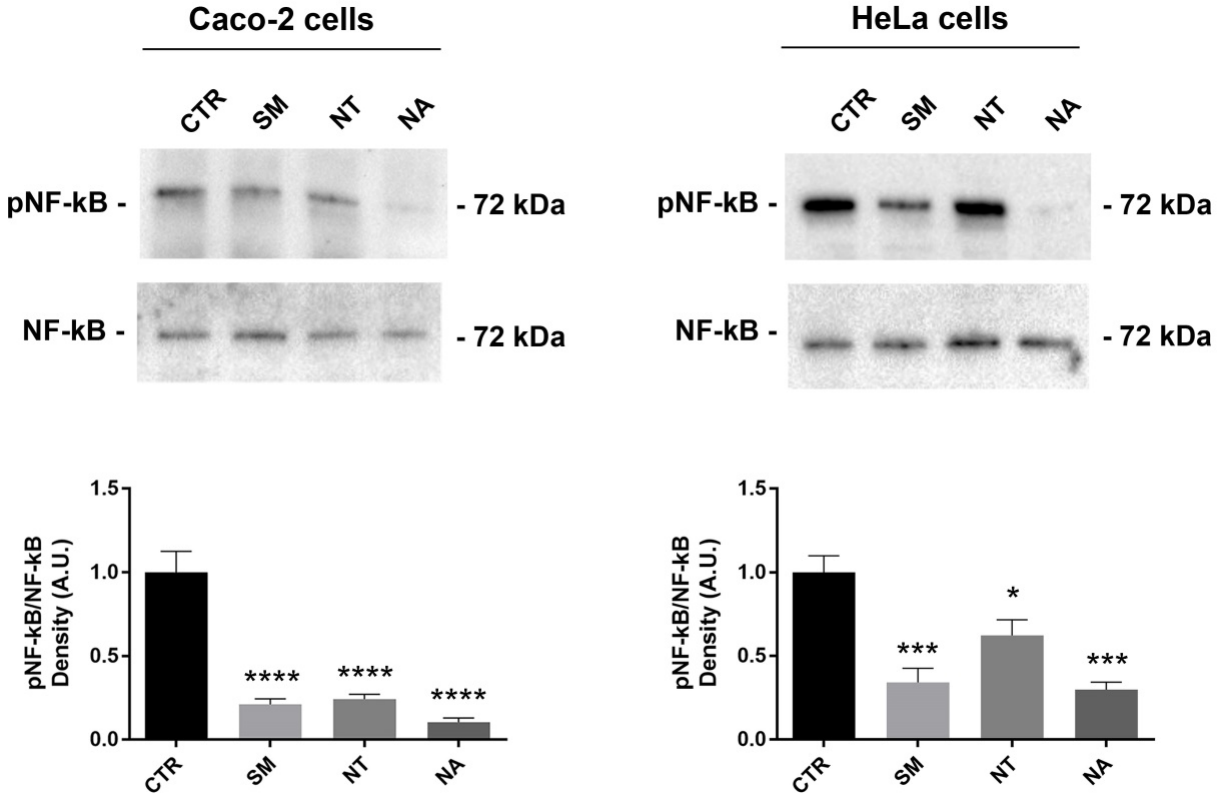
** Effects of non-alcoholic extracts obtained from SM, NT and NA on NF-κB phosphorylation in Caco-2 and HeLa cells.** Cells were treated as described in the methods section. An equal amount of proteins (60 µg/lane) was separated by gel electrophoresis and immunoblotted for evaluation of pNF-κB and NF-κB levels. Densitometry analysis of pNF-κB bands normalized to total NF-κB bands is reported in the histogram. Data are shown as means±SEM of 6-4 independent experiments and analyzed by One-way ANOVA followed by Dunnett's multiple comparisons tests (****p<0.0001, **p<0.01 and *p<0.05 *vs* CTR). Abbreviations: CTR, untreated conditions; NA, Negramaro; SM, Susumaniello; NT, Nero di Troia.

Flavonoids isolated from different plants exert antioxidant and anti-inflammatory effects in several cancer cell lines including breast and colon carcinoma cells by modulating intracellular signal pathways 50. The nuclear factor kappa B (NF-kB) is a key mediator of the inflammatory response. NF-kB activation requires the IkB ubiquitinylation and phosphorylation through IKK. Once activated by several stimuli, NF-kB translocates from the cytoplasm to the nuclei where it controls the expression of genes involved in inflammation, cell death, and proliferation 51. Several cancer cell lines, including HeLa cells, express the isoforms IKK-i/IKKϵ that control NF-kB activity by modulating the basal level of NF-kB (p65) phosphorylation at serine 536 that contributes to NF-kB function 52. Western Blotting studies revealed that treatment with non-alcoholic extracts obtained from SM, NT and NA significantly decreased the phosphorylation of NF-kB at serine-536 in Caco-2 and HeLa cells (Fig. 4). Together these findings suggested that these extracts down-regulate NF-kB function. Interestingly, several studies report that anthocyanins isolated from several plants including black rice and strawberry inhibit NF-kB activation 50,53,54. In bovine arterial endothelial cells, malvidin-3-glucoside downregulates pro-inflammatory factors by suppressing NF-kB function 55.

Besides NF-kB, several phytocompounds may affect certain intracellular signal transduction pathways involved in the inflammatory response, epithelial-to-mesenchymal transition (EMT), and cell death 56. In human glioma cells, piperlongumine stimulates p38-MAPK, increases IkB resulting in NF-kB downregulation 57.

**Figure 5:**
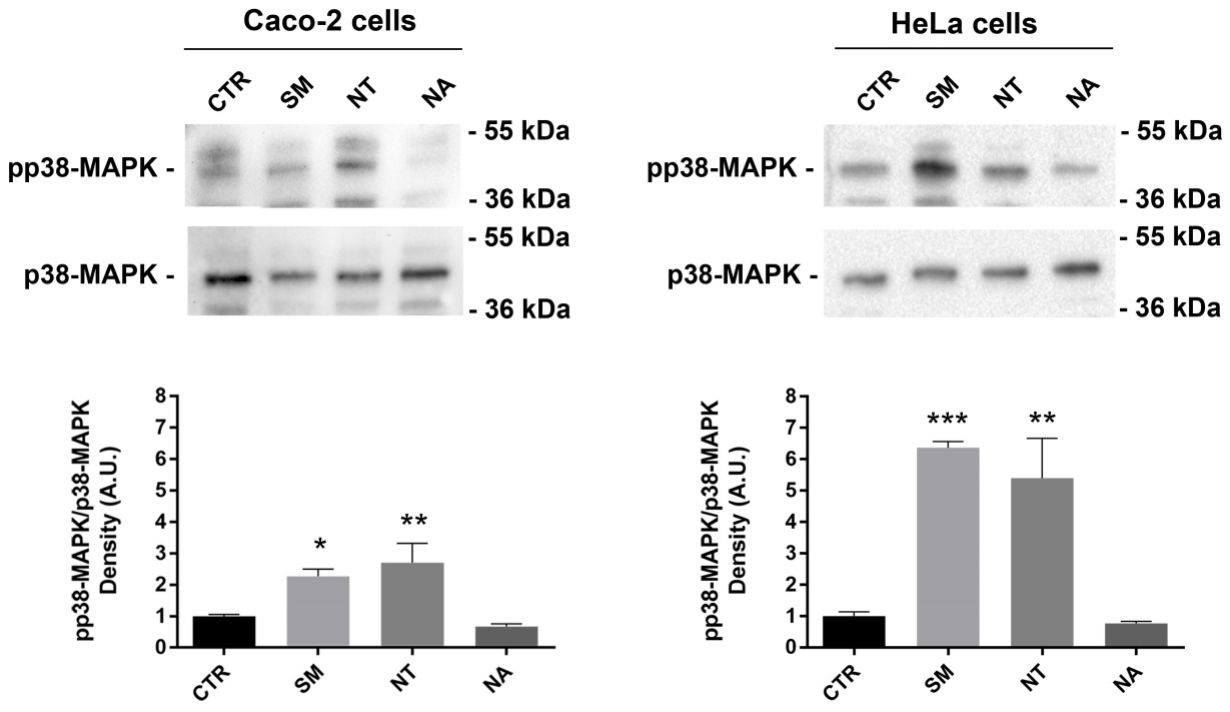
** Effects of non-alcoholic extracts on p38-MAPK phosphorylation in Caco-2 and HeLa cells.** An equal amount of proteins (60 µg/lane) was separated by gel electrophoresis and immunoblotted for evaluation of pp38-MAPK and p38-MAPK levels, as reported in the methods section. Densitometry analysis of pp38-MAPK bands normalized to total p38-MAPK bands is reported in the histogram. Data are shown as means±SEM of 4 independent experiments and analyzed by One-way ANOVA followed by Dunnett's multiple comparisons tests (****p<0.0001, **p<0.01 and *p<0.05 *vs* CTR). Abbreviations: CTR, untreated conditions; NA, Negramaro; SM, Susumaniello; NT, Nero di Troia.

Resveratrol, a natural polyphenol that is found in red wine, has a potent anti-tumor activity because it can induce apoptosis by activating p38-MAPK in non-small-cell lung cancer cells (NSCLC) and T-cell acute lymphoblastic leukemia cells 58,59. To investigate this signal pathway, a Western blot analysis was performed. Treatment with non-alcoholic wine extracts obtained from SM and NT resulted in a significant increase of the phosphorylation level of p38-MAPK compared with untreated cells (Fig. 5). No relevant effect on p38-MAPK phosphorylation was observed when cells were treated with the non-alcoholic extracts from NA.

In another study, however, it has been demonstrated that NA polyphenols activate p38-MAPK, downregulating NF-kB secondary to a significant increase of IKB-alpha phosphorylation 60. This discrepancy might be due to the different extraction procedures, the different concentrations of the extracts used in the previous study, as well as the different cell model. Moreover, in intestinal HT-29 cells, cyanidin-3-glucoside downregulates the inflammatory response by reducing the expression level of inflammatory cytokines without affecting p38-MAPK phosphorylation.

Taken together, these findings revealed that the non-alcoholic extracts isolates from different wines may differentially modulate multiple intracellular pathways. These effects might be related to the composition and the relative amounts of the single polyphenols in the extracts.

### Comparative transcriptomic analysis of grape berries

**Figure 6:**
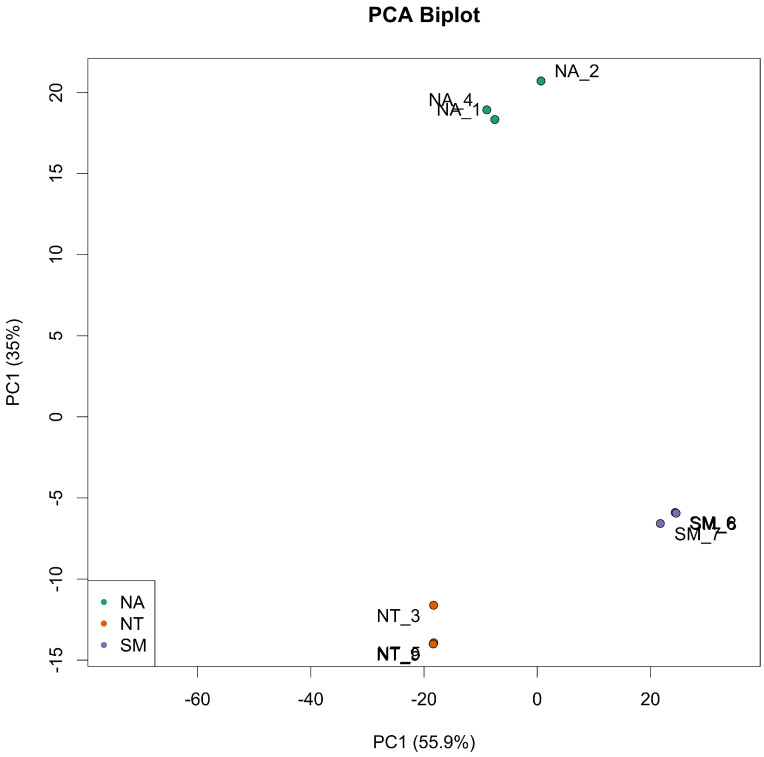
** Principal Component Analysis (PCA) of top 500 most variable genes.** Biological replicates are indicated as green dots for Negramaro samples (NA), red dots for Nero di Troia samples (NT) and purple dots for Susumaniello samples (SM).

Taking into account the *in vitro* assay results, three biological replicates of ripe berries from SM, NA and NT cultivars were harvested and subjected to a comparative transcriptome analysis using the RNA-seq technology. Nine libraries (NA1, NA2, NA4; NT3, NT5, NT9; SM6, SM7 and SM8) were prepared, each resulting in the production of an average 38 million paired-end reads through Illumina platform. After an appropriate cleaning procedure (see Material and Method Section), the fraction of reads uniquely mapping to the *V. vinifera* reference genome ranged from 90 to 95% (Tab. S3). The PCA analysis on the top 500 most variable genes showed a reduced or absent intra-sample variation but a remarkable inter-sample variation (Fig. 6).

**Figure 7:**
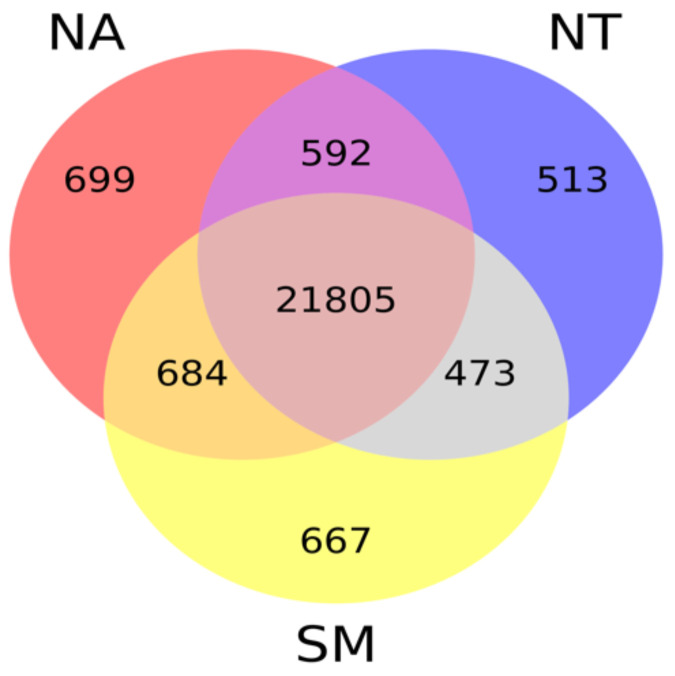
Venn diagram. The different circles represent the comparison between expressed genes in the three selected cultivars; numbers of unique and overlapping genes are reported. Abbreviations: NA, Negramaro; SM, Susumaniello; NT, Nero di Troia.

Taking into account gene expression values obtained for each cultivar, we found that the majority of known *V. vinifera* genes (21,805) were expressed in all selected samples while only a small fraction (whose number ranged between 513 and 699) was specifically expressed in each cultivar library (Fig. 7).

**Figure 8:**
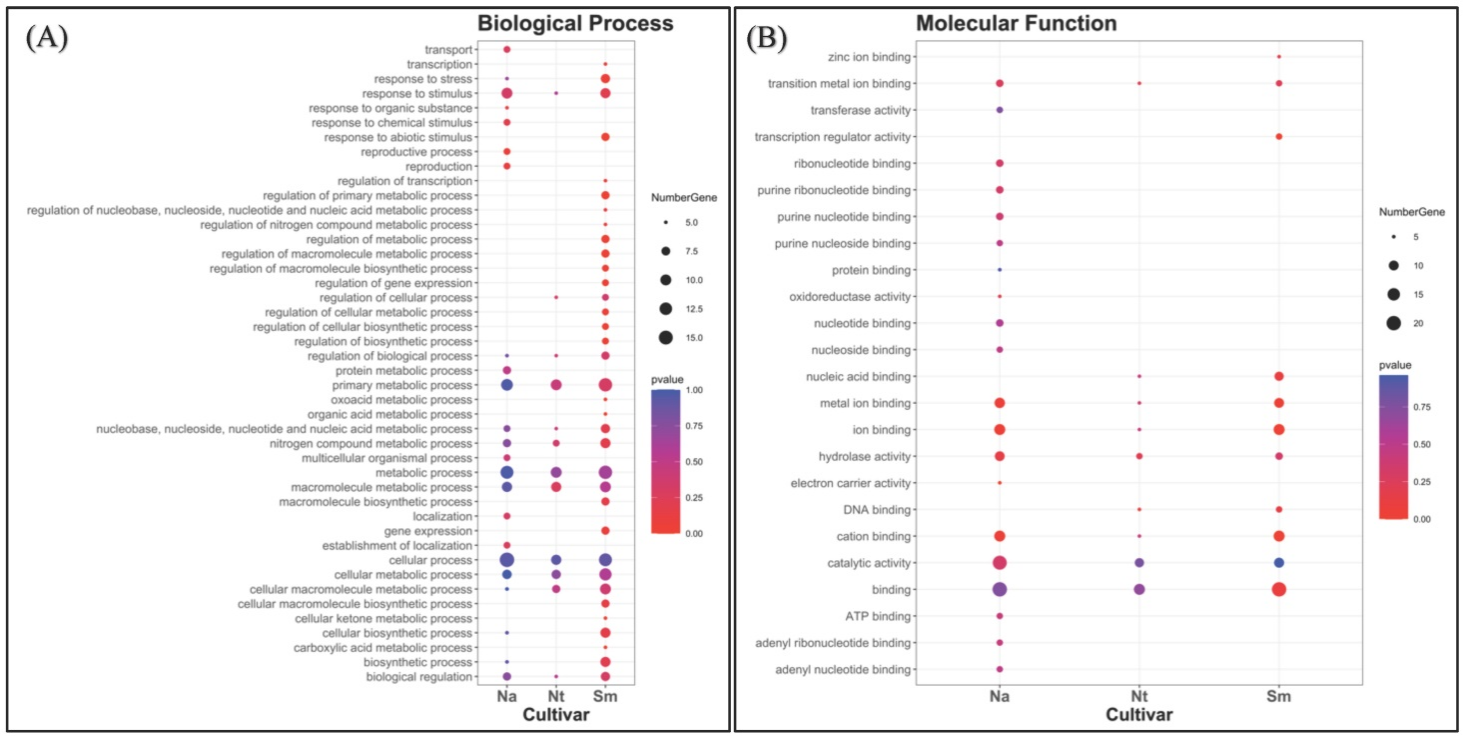
Classification of expressed unigenes in the investigated cultivars, according to biological process (**A**) and molecular function (**B**). Abbreviations: NA, Negramaro; SM, Susumaniello; NT, Nero di Troia.

To further characterize cultivar specific genes, we classified them according to two gene ontology categories, such as biological process (BP) and molecular function (MF) (Fig. 8). In general, the spectrum of BPs showed a trend of different complexity in the cultivars. While active processes were almost the same in NA and NT better overlapping each other, the SM spectrum was qualitatively different, mainly for some regulative processes (such as regulation of the cellular metabolic process, macromolecule biosynthetic process, gene expression, primary metabolic process, cellular process, etc.) (Fig. 8A). Genes related to the “metabolic processes” represented the major fraction in all the three populations, as well as genes related to the “cellular process” and “macromolecule metabolism process”. Moreover, little differences among the cultivars were detected about the “response to stimulus” category, which overall showed the highest number of genes in NA and SM grape berries.

The MF classification, instead, showed a more similar trend between NT and SM compared to NA (Fig. 8B). In particular, the SM spectrum contained the same GO categories of NT, plus two additional ones represented by “transcription regulator activity” and “zinc ion binding”, which were not even present in NA cultivar. Most of the unique molecular function categories present in NA was involved in the “nucleoside and nucleotide-binding”, while in all comparisons, the unigenes were categorized in large proportion as “involved in binding” and “catalytic activity”. These results are consistent with a still active metabolism in the collected berries.

**Figure 9:**
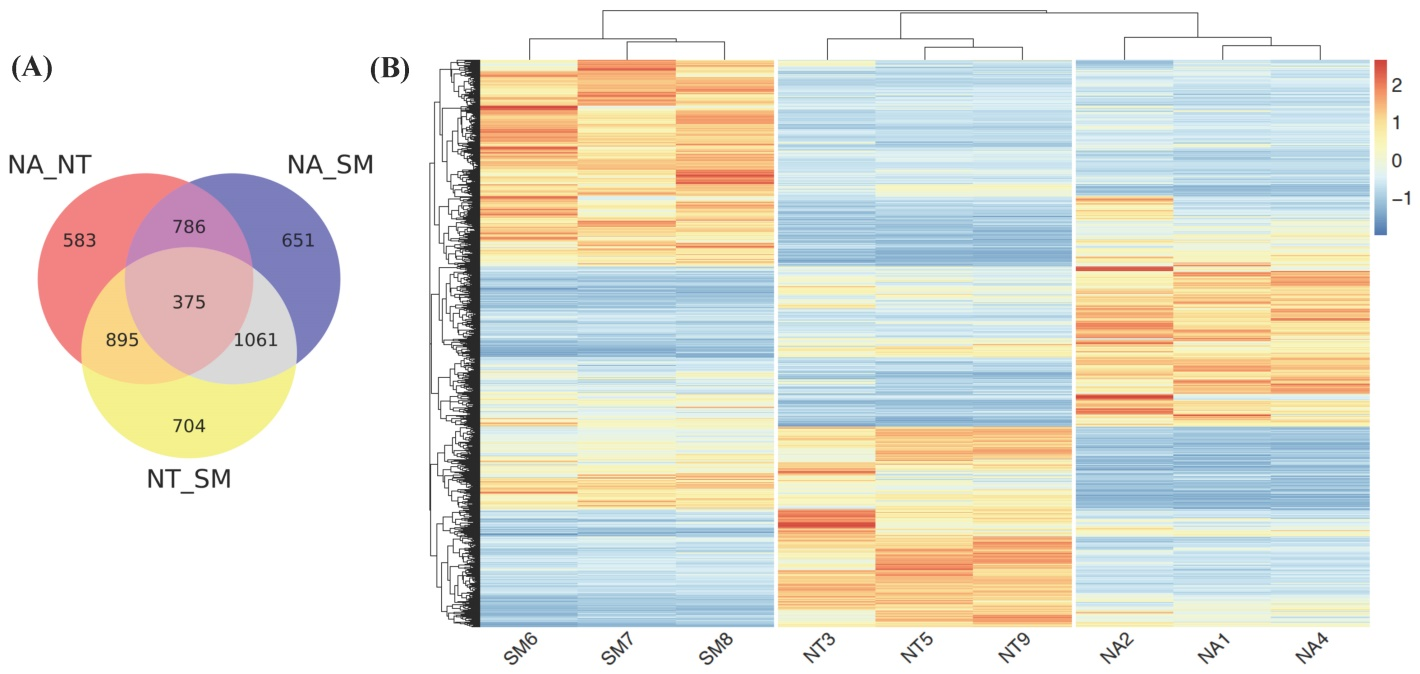
Differentially expressed genes (DEGs) analysis. **(A)** Venn diagram. The different circles represent a comparison between pairwise NA vs NT, NA vs SM, and NT vs SM. **(B)** Heat map related to DEGs in the three cultivars. Color intensity is proportional to the magnitude of changes. Relative expression levels are shown in red (up regulation) and blue (down regulation). Abbreviations: NA, Negramaro; SM, Susumaniello; NT, Nero di Troia.

We further investigated the differential gene expression in the three cultivars performing pairwise comparisons by means of DESeq2 (Fig. 9). Considering only genes with an adjusted *p* value <0.05 and |log_2_(FC)|>1, we found 2639, 2873 and 3035 DEGs in NA vs NT, NA vs SM and NT vs SM comparisons, respectively (Fig. 9A). The analysis of unique DEGs (5055) (Fig. 9B) revealed a clear and different distribution of DEGs in the three cultivars and the gene ontology classification showed the greatest variability of SM compared to NA and NT Fig. S1.

### Selection of candidate genes and expression analysis

**Figure 10:**
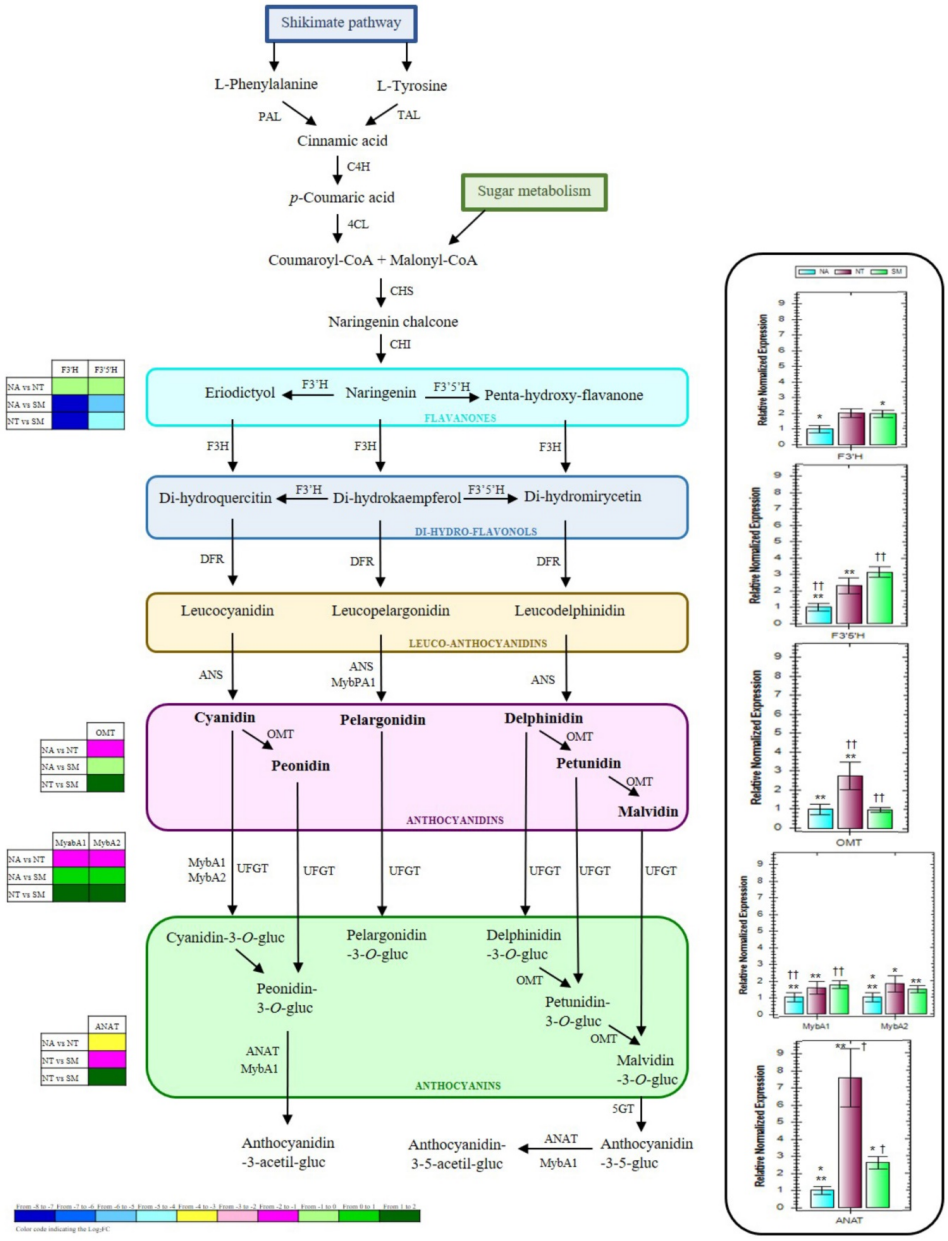
Simplified representation of the biosynthetic pathway of grape flavonoids, and relative expression levels of the selected genes (box on the right). The grids at the left show the gene expression profiles detected in NA, NT, and SM cultivars, as a result of the transcriptomic analysis. Results are mean values (± standard deviation) of three biological replicates. Negramaro has been set as a control sample (expression level = 1). Asterisks and crosses indicate significant pairwise differences between cultivars using Student's t-test (*,† and **, † † indicates *p* ≤ 0.05 and *p* ≤ 0.01, respectively). Abbreviations: PAL, Phenilalanine-ammonio-liase; TAL, Thirosin-ammonio-liase; C4H, Cinnamato-4-hydroxylase; 4CL, 4-cumarato-CoA-liase; CHS, Chalcone synthase; CHI, Chalcone isomerase; F3H, Flavanone-3-hydroxylase; F3'H, Flavanoid-3'-hydroxylase; F3'5'H, Flavanoid-3'5'-hydroxylase; DFR, Di-hydro-flavonol-4-reductase; ANS, Leucoanthocyanidin-dioxygenase; OMT, Anthocyanin-O-methyl-transferase; UFGT, UDP-glucose:flavonoid-3-O-glucosyl-transferase; 5GT, 5-glucosyl-transferase; ANAT, Acyl-transferase; MYBs, MYB transcription factors.

In accordance with DEG analysis and to obtain a global view on the biosynthetic pathway of grape flavonoids, and particularly of grape anthocyanins, KEGG pathway mapping tool, UNIPROT, and ENSEMBLPlants databases were used to classify the DEGs and highlight biological associations. Key proteins involved in the anthocyanin biosynthesis or into its genetic regulation were identified; thus, four structural (flavonoid-3'-hydroxylase, F3'H; flavonoid-3'5'-hydroxylase, F3'5'H; *O*-methyltransferase, OMT; and anthocyanin acyltransferases, ANAT) and two regulative genes (transcription factors MYBA1 and MYBA2) were considered worthy of attention and of further analysis. To better visualize the role of the selected proteins, in Figure 10 a simplified representation of the biosynthetic pathway of some important grape anthocyanins and the involved enzymes is reported. During grape berry veraison, many processes such as the biosynthesis and the accumulation of softening and aroma compounds start and generally continue until the berry maturation process is complete. In this process, genes involved in the biosynthesis of anthocyanins play a crucial role 10. Free anthocyanins in grape berries are synthesized via the flavonoid pathway, which shares the same upstream pathway with pro-anthocyanidins until the formation of anthocyanidins 61. In the early steps of this biosynthesis, the naringenin chalcone, derived by the condensation of three malonyl-CoA molecules with one *p*-coumaroyl-CoA molecule, can be successively modified to its isomer naringenin flavanone which consists of the typical basic three rings (C6-C3-C6) of the general flavonoid skeleton 62. The B ring of the naringenin can be further hydroxylated by a flavonoid-3'-hydroxylase (F3'H) or a flavonoid-3'5'-hydroxylase (F3'5'H) to form eriodyctiol or penta-hydroxy-flavanone. The oxidized form of naringenin, called dihydrokaempferol, could also be a potential substrate for these two enzymes, leading to the production of the corresponding di-hydro-flavonols. The temporal and tissue-specific expression of F3'H and F3'5'H in grape has been observed to be coordinated with the accumulation of the respective hydroxylated flavonoids, such as the anthocyanins 63. With extremely high stereospecificity, the di-hydro-flavonols are successively reduced to the corresponding colorless leucoanthocyanidins, and the latter to the corresponding anthocyanidins by the action of anthocyanidin synthase (ANS) (Fig. 10). Thus, the colored anthocyanidins are formed and immediately subjected to some modifications such as glycosylation, methylation, and acylation that guarantee their stability in the vacuole 61. The cytosolic *O*-methyltransferase (OMT) is usually responsible for the methylation of the hydroxyl groups at the C3' position or both at the C3' and C5' positions on the B rings of the anthocyanins 64. Previous studies reported its high expression level at berry veraison, highlighting the key role of this enzyme during grape anthocyanin biosynthesis 65. Anthocyanin acylation, represented by the addition of aromatic and/or aliphatic constituents, is also one of the most common and important modifications, that can greatly enhance the structural diversity, color stabilization, and intensity as well as the shift to blue color of these compounds 66. The acylation reactions are catalyzed by the so-called anthocyanin acyltransferases (ANAT) (Fig. 10), which usually have high substrate specificity for both the anthocyanin acceptors and the acyl group donors 67.

In red grapes, the anthocyanin accumulation has been reported to be influenced by several factors, including genetic and phytohormonal regulations and viticultural practices 61,68. Indeed a complex network of multiple regulatory genes, mainly belonging to the Myb and Myc transcription factor families and to the WD40-like proteins, is involved at the transcriptional level 69-73. In particular, the downstream steps leading to anthocyanin formation through glycosylation and subsequent modifications (methylation and acylation) are under the specific control of several R2R3-Myb factors, belonging to the VvMybA gene cluster 74-76 (Fig. 10). Among them, the VvMybA1 and VvMybA2 have been recognized among the principal regulators of anthocyanin accumulation in grape berries, since mutations for these genes caused white-fruited grapes 77,78.

In this study, the expression profiles of F3'H, F3'5'H, OMT, ANAT, MYBA1, and MYBA2 genes were investigated by qRT-PCR analysis (Fig. 10). The lowest expression levels of F3′H and F3′5′H were generally observed in NA. On the other hand, NT and SM cultivars showed comparable expression profiles for F3′H gene, while the highest expression value of F3′5′H gene was observed only in SM (Fig. 10). Both OMT and ANAT genes resulted over-expressed in NT, whereas OMT gene reached an almost triple value in comparison with those observed in the other two cultivars, while ANAT gene reached values up to seven and three times higher when compared with NA and SM, respectively. Concerning the regulation genes MYBA1 and MYBA2, the transcript levels resulted significantly rather low in NA than in the other cultivars (Fig. 10).

Thus, the overall expression trend of the selected genes related to anthocyanin biosynthetic pathway was generally higher in NT and SM cultivars, with comparable expression levels for F3'H, F3'5'H, MYBA1, and MYBA2, and significantly very different levels for OMT and ANAT. For most of the considered genes, the qRT-PCR results were in accordance with the RNA-Seq data.

## Conclusions

As support to the current availability of autochthonous cultivars in the official productive panorama, the Apulian viticulture retains a huge biodiversity heritage. In this work we evaluated the total phenol and anthocyanin content in five autochthonous Apulian grapevine cultivars, in both wines and their non-alcoholic extracts. On one hand, the cultivars P, NA and NT represent the most famous and important Apulian autochthonous grapevines; on the other hand, several minor grapevine varieties, such as SM and N, belonging to known genotypes or with a unique fingerprinting, are recently and powerfully entering the market, with stable production and new important agronomic traits.

The potential antioxidant effects of the non-alcoholic wine extracts on the cell viability of Caco-2 and HeLa carcinoma cell lines were also tested. Finally for the most promising autochthonous selected cultivars (NA, NT and SM), a comparative transcriptomic analysis in berries was performed using high-throughput sequencing technology.

In conclusion, the detailed characterization of these cultivars and their derived products at chemical, physiological and molecular levels, could contribute to widening the range of new commercial proposals increasing the competitiveness and improving the entire Apulian wine-making chain. The obtained results showed how the genetic background of grape cultivar plays a key role on the quality of bioactive compounds in derived red wines. In this contest, the importance to valorize and characterize minor cultivars would be extremely relevant to propose new products with benefits on human health.

## Supplementary Material

Supplementary figure and tables.

## Figures and Tables

**Table 1:** Chemical characterization of wines

Parameters	NA	N	P	SM	NT
pH	3.52	3.51	4.27	3.77	3.61
Alcohols (% v/v)	11.85	11.64	13.02	11.84	11.82
Glycerol (g/L)	11.47	9.02	17.97	9.06	8.71
Methanol (g/L)	0.20	0.21	0.36	0.19	0.17
Sugars (g/L)	1.59	2.75	4.32	1.93	0.94
Titratable acidity (g/L)	7.54	9.10	7.73	5.62	6.86
Volatile acidity (g/L)	0.41	0.52	0.52	0.54	0.51
Ashes (g/L)	2.58	2.24	4.82	2.67	2.64
K^+^ (g/L)	1.32	0.98	2.18	1.06	1.04
Dry residue extract (g/L)	31.82	30.63	55.64	29.68	26.92

Mean values of 2 batches are reported. Abbreviations: NA, Negramaro; N, Notardomenico; P, Primitivo; SM, Susumaniello; NT, Nero di Troia.

**Table 2:** Total phenol content and anthocyanin profiles in non-alcoholic extracts

Parameters	NA		N		P		SM		NT	
Total phenol content	1301	±58 c	1968	±46 bc	5824	±354 a	2856	±102 b	5791	±349 a
Delfinidin-3-glu	32.3	±1.3 c	49.5	±0.1 a	10.3	±1.2 d	42.0	±0.1 b	32.5	±0.7 c
Cyanidin-3-glu	6.9	±0.4 b	12.5	±1.2 a	3.8	±0.1 c	6.0	±0.5 bc	6.5	±0.2 b
Petunidin-3-glu	60.0	±2.9 b	55.9	±0.1 b	10.8	±0.5 d	77.0	±0.7 a	43.0	±0.3 c
Peonidin-3-glu	14.9	±0.7 b	51.6	±2.8 a	5.3	±0.1 c	45.4	±3.5 a	19.0	±0.8 b
Malvidin-3-glu	280.0	±20.7 b	191.9	±0.8 c	170.7	±5.4 c	570.8	±1.5 a	272.1	±7.2 b
Malvidin-3-acetylglu	2.4	±0.1 c	3.1	±0.1 c	2.3	±0.2 c	12.8	±0.6 a	4.6	±0.3 b
Malvidin-3-coumarylglu	11.1	±0.6 d	9.8	±0.1 d	13.7	±0.3 c	58.2	±0.2 a	41.0	±0.7 b

The results are expressed as mean of 2 batches ± standard deviation (mg/L). Different letters indicate statistical differences at *p*<0.05 determined by ANOVA and Tukey's test. Abbreviations: NA, Negramaro; N, Notardomenico; P, Primitivo; SM, Susumaniello; NT, Nero di Troia; glu, glucoside; acetyglu, acetylglucoside; coumarylglu, coumarylglucoside.
