# Supplementary material for: “Good Wine Makes Good Blood”: An Integrated Approach to Characterize Autochthonous Apulian Grapevines as Promising Candidates for Healthy Wines
Source: Int J Biol Sci. 2022 Apr 11;18(7):2851–66. doi: 10.7150/ijbs.70287 (PMC9066116; doi:10.7150/ijbs.70287)

SUPPLEMENTARY MATERIALS

"Good Wine Makes Good Blood": An Integrated Approach To Characterize Autochthonous Apulian Grapevines As Promising Candidates For Healthy Wines

Wilma Sabetta[1,2*], Mariangela Centrone[3], Mariagrazia D'Agostino[3], Graziana Difonzo[4], Luigi Mansi[3], Giovanni Tricarico[5], Pasquale Venerito[6], Ernesto Picardi[3], Luigi Ruggiero Ceci[7], Grazia Tamma[3], Francesco Caponio[4], Cinzia Montemurro[2,4,8*], Mariateresa Volpicella[3*]

1) Institute of Biosciences and BioResources (IBBR), National Research Council (CNR), Via Amendola 165/A, 70126 Bari, Italy;
2) Spin off Sinagris.r.l., University of Bari Aldo Moro, Via Amendola 165/A, 70126 Bari, Italy;
3) Department of Biosciences, Biotechnologies and Biopharmaceutics, University of Bari Aldo Moro, Via Amendola 165/A, 70126 Bari, Italy;
4) Department of Soil, Plant and Food Sciences, University of Bari Aldo Moro, Via Amendola 165/A, 70126 Bari, Italy;
5) Confcooperative Puglia, Viale Einaudi 15, 70125 Bari, Italy;
6) CRSFA-Centro Ricerca, Sperimentazione e Formazione in Agricoltura "Basile Caramia", Via Cisternino, 281, 70010 Locorotondo (BA), Italy;
7) Institute of Biomembranes, Bioenergetics and Molecular Biotechnologies, National Research Council (CNR), Via Amendola 165/A, 70126 Bari, Italy;
8) Institute for Sustainable Plant Protection–Support Unit Bari, National Research Council (IPSP-CNR), Via Amendola 165/A, 70126 Bari, Italy.

*Correspondence: wilma.sabetta@ibbr.cnr.it; cinzia.montemurro@uniba.it; mariateresa.volpicella@uniba.it

Table S1: Untrimmed and trimmed sequences for the different cultivars.

Table S2: Paired-end (PE) and mapped reads, with relative percentage, obtained after sequence alignment.

Table S3: Target genes and specific primer pairs used for qRT-PCR.

Figure S1: Classification of expressed DEGs in the investigated cultivars between pairwise NA vs NT, NA vs SM and NT vs SM, according to molecular function (MF) and biological process (BP).

Table S1. Untrimmed and trimmed sequences for the different cultivars. Biological replicates are indicated in the sample-id column as NA for Negramaro cv, NT for Nero di Troia cv and SM for Susumaniello cv.

Sample-id	Untrimmed sequences	Trimmed sequences
**NA_1**	72.474.666	64.278.602
**NA_2**	83.498.082	73.995.480
**NA_4**	93.439.632	83.434.718
**NT_3**	73.514.034	64.894.852
**NT_5**	72.558.438	64.219.248
**NT_9**	77.299.542	66.473.998
**SM_6**	67.765.598	59.719.112
**SM_7**	72.167.418	63.647.554
**SM_8**	78.083.724	67.947.888

Table S2. Paired-end (PE) and mapped reads, with relative percentage, obtained after sequence alignment.Biological replicates are indicated in the sample-id column as NA for Negramaro cv, NT for Nero di Troia cv and SM for Susumaniello cv.

Sample-id	Input reads (PE)	Mapped reads	%
**NA_1**	32.139.301	30.137.507	93.77
**NA_2**	36.997.740	33.136.426	89.56
**NA_4**	41.717.359	39.401.207	94.45
**NT_3**	32.447.426	30.773.205	94.84
**NT_5**	32.109.624	30.408.021	94.70
**NT_9**	33.236.999	31.327.726	94.26
**SM_6**	29.859.556	28.004.595	93.79
**SM_7**	31.823.777	29.684.781	93.28
**SM_8**	33.973.944	31.808.945	93.63

Table S3. Primer sequences for qRT-PCR analysis.

Gene		Primer sequence (5'→3')	Amplicone size (bp)	Reference
VvF3'H	Fw	GAGAAGAGGTGGACGGAGCAAATC	167	Zhang et al., 2018
	Rv	GCCTCCGTTGCCGCTCAGTT		
VvF3'5'H	Fw	AAACCGCTCAGACCAAAACC	100	Azuma et al., 2012
	Rv	ACTAAGCCACAGGAAACTAA		
VvOMT	Fw	CTCTGCAGGCGCCTCTATTA	139	Cutanda-Perez et al., 2009
	Rv	CCCAAAACAGAGTCTGGACA		
VvANAT	Fw	CACCATTACCAGGCATTT	120	Sun et al., 2016
	Rv	CCTCCCTTAGTAGACCCAC		
VvMybA1	Fw	GTCCTTGATTGCGGGTAG	149	Sun et al., 2016
	Rv	GAGGCTTTATAGCTTTGGTT		
VvMybA2	Fw	CCAGTAAGCCATCATCCACG	140	by authors
	Rv	TGTCCAGAGGCTTGCGATAA		
VvEF1γ	Fw	CAAGAGAAACCATCCCTAGCTG	92	Hichri et al., 2010
	Rv	TCAATCTGTCTAGGAAAGGAAG		

Figure S1: Classification of expressed DEGs in the investigated cultivars between pairwise NA vs NT, NA vs SM and NT vs SM, according to molecular function (MF) and biological process (BP). Abbreviations: NA, Negramaro; SM, Susumaniello; NT, Nero di Troia; UP, Upregulated DEGs; DOWN, Downregulated DEGs.

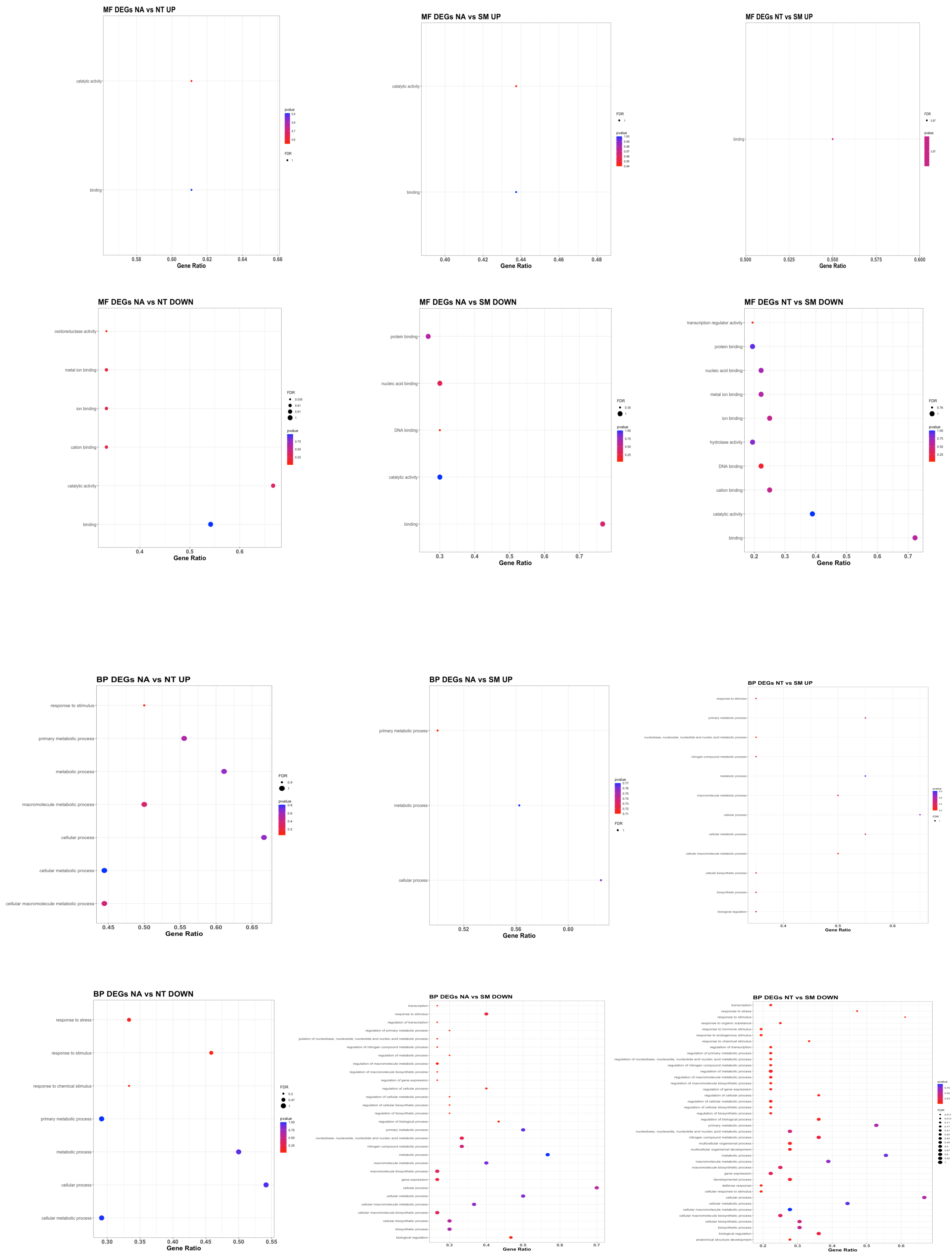

Supplement: Supplementary file 1 — Supplementary figure and tables. [file ijbsv18p2851s1.pdf]
